# Risk Management Assessment Improves the Cost-Effectiveness of Invasive Species Prioritisation

**DOI:** 10.3390/biology10121320

**Published:** 2021-12-12

**Authors:** Peter A. Robertson, Aileen C. Mill, Tim Adriaens, Niall Moore, Sonia Vanderhoeven, Franz Essl, Olaf Booy

**Affiliations:** 1 Modelling, Evidence and Policy Group, Natural and Environmental Sciences, Newcastle University, Newcastle upon Tyne NE1 7RU, UK; aileen.mill@ncl.ac.uk (A.C.M.); olaf.booy@apha.gov.uk (O.B.); 2 Research Institute for Nature and Forest (INBO), Havenlaan 88 Bus 73, B-1000 Brussel, Belgium; tim.adriaens@inbo.be; 3 GB Non-Native Species Secretariat, Animal and Plant Health Agency, Sand Hutton, York YO41 1JW, UK; niall.moore@apha.gov.uk; 4 Belgian Biodiversity Platform, Walloon Research Department for Nature and Agricultural Area (DEMNA), Service Public de Wallonie, Avenue Maréchal Juin, 23, B-5030 Gembloux, Belgium; s.vanderhoeven@biodiversity.be; 5 Bioinvasions Global Change, Macroecology-Group, Department of Botany and Biodiversity Research, University of Vienna, Rennweg 14, 1030 Vienna, Austria; franz.essl@univie.ac.at

**Keywords:** Aichi Target 9, Europe, invasive alien species of Union Concern, management cost, management feasibility, prioritisation, risk assessment, risk management

## Abstract

**Simple Summary:**

International agreements commit nations to control or eradicate invasive alien species. The scale of this challenge exceeds available resources and so it is essential to prioritise the management of invasive alien species. Species prioritisation for management may consider the likelihood and scale of impact (risk assessment) and the feasibility, costs and effectiveness of management (risk management). Risk assessment processes are widely used, risk management less so. To assess the cost effectiveness of prioritisation, we considered 26 high-risk species considered for eradication from Great Britain (GB) with pre-existing risk assessment and risk management outputs. We used these to consider the relative reduction in risk per unit cost when managing prioritised species based on different criteria. We showed that the cost effectiveness of prioritisation within our sample using risk assessment scores alone performed no better than a random ranking of the species. In contrast, prioritisation including management feasibility produced nearly two orders of magnitude improvement compared to random ranking. We concluded that basing management actions on priorities based solely on risk assessment without considering management feasibility risks the inefficient use of limited resources. In this study, the cost effectiveness of species prioritisation action was greatly increased by the inclusion of a risk management assessment.

**Abstract:**

International agreements commit nations to control or eradicate invasive alien species. The scale of this challenge exceeds available resources and so it is essential to prioritise the management of invasive alien species. Species prioritisation for management typically involves a hierarchy of processes that consider the likelihood and scale of impact (risk assessment) and the feasibility, costs and effectiveness of management (risk management). Risk assessment processes are widely used, risk management less so, but are a crucial component of resource decision making. To assess the cost-effectiveness of prioritisation, we considered 26 high-risk species considered for eradication from Great Britain (GB) with pre-existing risk assessment and risk management outputs. We extracted scores to reflect the overall risk to GB posed by the species, together with the estimated cost and the overall feasibility of eradication. We used these to consider the relative reduction in risk per unit cost when managing prioritised species based on different criteria. We showed that the cost-effectiveness of prioritisation within our sample using risk assessment scores alone, performed no better than a random ranking of the species. In contrast, prioritisation including management feasibility produced nearly two orders of magnitude improvement compared to random. We conclude that basing management actions on priorities based solely on risk assessment without considering management feasibility risks the inefficient use of limited resources. In this study, the cost-effectiveness of species prioritisation for action was greatly increased by the inclusion of risk management assessment.

## 1. Introduction

Managing the increasing risks and impacts of invasive alien species (IAS) is one of the great societal challenges of the 21st century [1,2,3,4,5]. A number of ambitious international goals aim to reduce or halt the rising impacts of alien species. For instance, Aichi Target 9 of the Convention on Biological Diversity [6] aims to substantially reduce the loss of biodiversity and commits signatories to identify and prioritise IAS and their pathways and to then control or eradicate priority species, a commitment that carries significant economic and social costs. The European Union Regulation (EU)1143/2014 on Invasive Alien Species [7,8] reflects these goals [9]. 

International and national legislation on IAS and their supporting processes are still evolving [10], while the resources available for action remain small compared to the scale of the challenge [11]. The prioritisation of species, pathways and management is a key element of international targets and should support decision making to achieve the cost-effective management of IAS. An appropriate metric underpinning prioritisation is the greatest reduction in impact on biodiversity, ecosystem services and human interests per unit cost. Studies have examined the relative cost effectiveness of interventions at different stages in the invasion process, highlighting the benefits of prevention and rapid response compared to on-going management [12,13,14,15]. However, as national strategies emerge, there is a need for studies to compare the cost effectiveness of different approaches to the prioritisation of species or the effects of including different criteria on the outcome. 

A range of existing processes is available to support prioritisation based on different criteria. These may be used in isolation or in a sequential manner, and their use varies between different countries. A risk assessment (RA) is a systematic approach to assess the scale and likelihood of arrival, establishment, spread and impact of alien species to identify those that are likely to become invasive [16,17,18,19,20,21,22]. Different forms of RA include the rapid horizon scanning identification of species that may pose risks in future [20] through to formal detailed assessments of individual species that may underpin trade restrictions and legislation. The approach is further developed through the standardised assessment of species’ impacts (a major component of RA) [17,18,23]. Studies offering prioritised lists of IAS based on RA have a high policy profile, including lists of the ‘worst’ IAS [24,25,26], and RA is the main evidence underpinning the listing of Species of Union Concern in Europe. There are calls for these RA-based approaches to be more widely applied to species listing [27,28]. 

A further set of methods considers the feasibility and costs of management. These are often applied subsequent to the initial identification of high-risk species through RA. We collectively refer to these approaches as risk management (RM), although other terms, such as risk treatment, are also used. They provide the process by which the cost–benefit [29] or feasibility of taking action [30] can be assessed in a standardised manner. For IAS, RM includes the assessment of the practical, resource, social, ethical, political and legal constraints under which management must occur. It places these in the context of the biological characteristics of the species [31], its stage in the invasion process, the scale of the problem, as well as the socio-economic and ecological costs and consequences of its management [22,30]. RM principles have been applied to government-led invasive species programs in countries such as Australia and New Zealand since the early 2000s [32,33], while the same process has been applied in a number of discrete studies [30,31,34]. However, the use of this approach remains sporadic and specific to particular countries or regions. For example, the listing of Species of Concern within The European Union does not include any formal assessment of management feasibility [35]. 

Effective prioritisation of species and management options are essential if the limited funds available to reduce the impacts of IAS are to be effectively targeted. Given the variety of approaches applied in different countries, there is a need to better understand the cost effectiveness of different approaches to the prioritisation of species and their management, together with the effects of including different criteria on the outcome. 

Here, we consider the cost effectiveness of different approaches to prioritisation for rapid eradication based on a sample of high-risk species already identified as having high potential impacts in Great Britain. We compare the cost effectiveness of prioritisation based on the use of RA, RM and both methods in combination, using scores from independently undertaken RA and RM assessments already published in the literature. We use a simple cost effectiveness measure of cumulative risk reduction per unit cost to compare different approaches to prioritisation and attempt to quantify the benefits of incorporating both RA and RM considerations in decision making and risk communication through a wider process of risk analysis. 

## 2. Materials and Methods

We assessed a total of 26 species considered likely to establish in GB in the near future or that were already established, but with limited distributions. The 15 established species were (see Supplementary Materials): *Alopochen aegyptiacus* (Egyptian goose), *Cabomba caroliniana* (a fanwort), *Dreissena bugensis* (quagga mussel), *Egeria densa* (large-flowered waterweed), *Hemigrapsus sanguineus* (Asian shore crab), *Hydropotes inermis* (Chinese water deer), *Ichthyosaura alpestris* (alpine newt), *Lacerta bilineata* (green lizard), *Lysichiton americanus* (American skunk-cabbage), *Orconectes limosus* (spiney-cheek crayfish), *Orconectes virilis* (virile crayfish), *Podarcis muralis* (wall lizard), *Procambarus acutus* (white river crayfish), *Procambarus clarkia* (red swamp crayfish) and *Sarracenia purpurea* (purple pitcher plant). The 11 species considered likely to establish in the near future were: *Corbicula fluminalis* (Asian clam), *Corvus splendens* (house crow), *Gracilaria vermiculophylla* (a seaweed), *Homarus americanus* (American lobster), *Mnemiopsis leidyi* (American comb jelly), *Nyctereutes procyonoides* (raccoon dog), *Procyon lotor* (raccoon), *Rapana venosa* (rapa whelk), *Tamias sibiricus* (Siberian chipmunk), *Threskiornis aethiopicus* (sacred ibis) and *Vespa velutina* (Asian hornet). Together these 26 species comprise 5 plants, 11 invertebrates and 10 vertebrates from marine (5), freshwater (9) and terrestrial (12) environments. RA and RM scores were available for all 26 species from previous studies, the methods and results of which are publicly available [20,30,36].

RA scores for each species included an overall risk score, as well as four component scores representing the separate risks of entry, establishment, spread and species impact from published risk assessments [36]. Each component was scored from 1–5 (very unlikely/minimal–very likely/massive), while overall risk was scored from 1–3 (low–high).

RM scores for each species included a score for the overall feasibility of eradication, and seven component scores for effectiveness, practicality, management cost, management impact, acceptability, likelihood of reinvasion and window of opportunity [29]. Each component and the overall feasibility of eradication was scored from 1–5 (very low–very high, except for the component cost, which was scored 1—< GBP 50 k, 2— GBP 50–200 k, 3— GBP 200 k to GBP 1 M, 4— GBP 1 M to 10 M, 5— GBP 10 M+).

We estimated the cost of eradicating each species as the mid-point of the RM component ‘cost’ (e.g., for a cost of GBP 200 k–1 M a mid-point of GBP 600,000 was used). The potential benefit of eradication was considered to be the impact removed by eradicating each species and was calculated by multiplying the RA components ‘spread’ (1–5) and ‘impact’ (1–5), resulting in a total potential impact removed score of 1–25. Spread and impact were combined because taken together they assess both the extent and severity of potential impact.

Overall, RA and RM scores were used to order species using three different prioritisation methods:

- RA = overall risk, from 3 (high risk) to 1 (low risk);
- RM = overall feasibility of eradication, from 5 (very high) to 1 (very low); and,
- RA + RM = sum of numeric scores for overall risk and overall feasibility of eradication, from 8 (highest) to 2 (lowest). Note that this method is the equivalent of the matrix approach used in previous presentations of this data [30].

Ties within both the RA and RM methods were ordered by the geometric mean of the individual component scores (for RA component scores; entry, establishment, spread and impact. For RM component scores; effectiveness, practicality, cost, impact and acceptability). Ties within the RA + RM method were ordered by the highest sum of the geometric mean of RA and RM component scores. 

The relative cost–benefit of these three prioritisation methods was examined by comparing the cumulative cost of eradicating species in prioritised sequence to the cumulative benefit of removing their potential impact. Comparison was made using the area under the curve (AUC) statistic [37], where a greater AUC had a higher cost–benefit. Statistical significance and confidence intervals were assessed by permutation of the IAS list to generate 10,000 randomised species rankings and AUC values using the R package simctest [38].

## 3. Results

**Figure 1:**
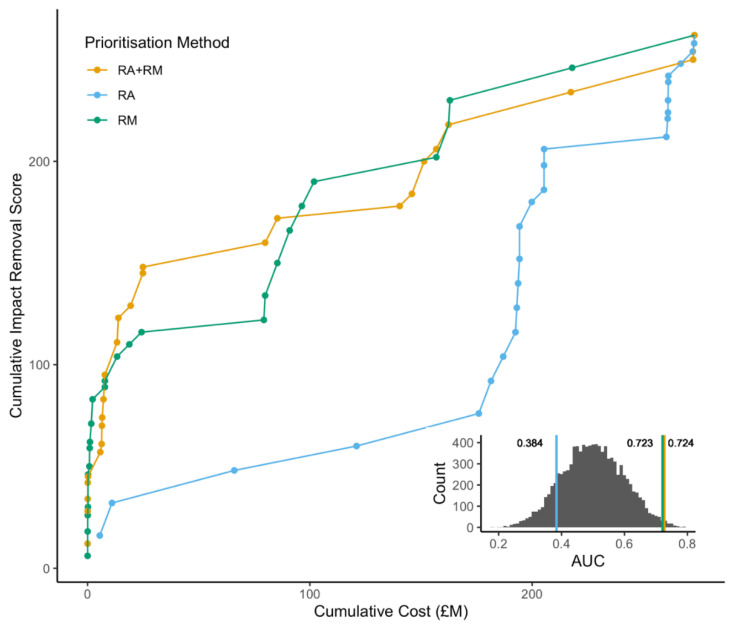
Cost–benefit curves of three prioritisation approaches based on risk assessment (RA, blue), risk management (RM, green) or a combination of both (RA + RM, yellow). Points denote the cumulative cost (GPB M) and impact reduction for the removal of species on each prioritised list in rank order. The area under the curve (AUC) for each approach was calculated, and significance was assessed by 10,000 permutations. The inset shows the frequency distribution of AUC from 10,000 randomised IAS lists compared to the prioritisation approaches. (Original data on risk assessment and risk management scores from previously published studies [20,30,36]).

The cost–benefit of eradicating species prioritised by RM and RA + RM methods had a significantly higher AUC than randomised lists (RM; AUC = 0.723, the 95% confidence interval (CI) for the *p*-value under the null hypothesis was (0.005–0.032), RA + RM; AUC = 0.724, *p*-value 95% CI (0.005–0.032)). The RA prioritisation gave an AUC less than the mean of the random rankings (RA; AUC = 0.384, *p*-value 95% CI (0.358–0.994)) (Figure 1).

The cost of eradicating the top ten ranked species identified by the different approaches to prioritisation varied by nearly two orders of magnitude, with priorities identified by RM and RA + RM providing a significantly greater reduction in risk per unit cost than the use of RA alone (Table 1).

## 4. Discussion

Our analysis shows including the evaluation of risk management in IAS prioritisation yields greater risk reduction per unit cost. Prioritising species present in Great Britain with limited distributions or those likely to establish in the near future by risk assessment alone favoured species that were more costly to eradicate. While preventative methods might also be considered to limit the arrival of IAS, rapid eradication was a valid management option for such species, so we consider this to be a realistic assessment of the species prioritisation choices required at this stage of the invasion process in GB.

There was little correspondence between the lists produced by RA alone or RM/RA + RM, with only one species in the top ten RA ranking featured in the RM or RA + RM lists, while the RM and RA + RM ranks contained nine shared species in the first ten. The RA approach in isolation prioritised three species that were considered very costly to eradicate and where the overall feasibility of management was scored low or very low. The cost of eradicating the top ten species prioritised through the use of RM or RA + RM was two orders of magnitude lower than if it were based on RA alone, and the cost effectiveness of the prioritisation of management based on RA alone offered no improvement over selecting species in a random order. 

This study used existing data from previous studies [20,30,36]. These assessed RA and RM using the available literature combined with expert opinion to score species in relative terms. While this is appropriate when considering the relative risks and feasibility of management, these papers recommend more detailed assessment of the case-specific costs and benefits of management [13,14,15] before reaching a final conclusion. This same caveat applies to the results of this analysis, for which prioritisation identifies species for more detailed consideration.

This example is based on the prioritisation for the rapid eradication of species with a limited distribution. We expect that the relative value of RA and RM varies depending on the stage of the invasion process, and the processes are often used in a hierarchical sequence. RA is particularly useful for horizon scanning to identify species that may become invasive and to inform management to prevent their entry or establishment. When considering rapid removal, eradication and long-term management [39] to deal with species once they have entered and the scale of the problem becomes an issue, then RM becomes increasingly important to ensure that actions are feasible and resources are used cost effectively. Indeed, we would expect the improved cost effectiveness of combining RA and RM to be significantly greater than described here when considering the eradication or on-going management of species that are already widespread. Calls to increase the use of RA to assess species that are already widespread would benefit from the inclusion of RM considerations [27,28], particularly if they led to the inclusion of new management responsibilities on European member states. 

The use of systematic methods to guide IAS legislation and action has clear benefits. RA is a key element of this, but if used to prioritise species for management in isolation it can lead to a mismatch between the species being prioritised and the most cost-effective approaches to management. Few published lists of priority IAS consider management feasibility, and lists such as the EU Species of Union Concern are based on RA, although the inclusion of a species on these lists places management responsibilities on the EU member states. Here, we show that the inclusion of RM as part of a broader risk analysis process for the prioritisation of IAS significantly improved cost effectiveness compared to the use of RA alone. We believe that RA and RM should be used together to guide the species priorities, in particular when considering eradication and on-going management action [5,39]. This is the approach already used to manage risks in some regions [32,33] and other sectors dealing with biosecurity, such as the Food and Agriculture Organization (FAO) for plant health [40,41], the World Organisation for Animal Health (OiE) and the World Health Organization for human health (WHO) [42,43], where decision making around prioritisation for action is based on a wider process of risk analysis, consisting of risk assessment and risk management, together with subsequent risk communication. 

## 5. Conclusions

The efficient prioritisation of invasive alien species for management is important if limited resources are to be deployed effectively to reduce their impacts. We showed that using only risk assessment scores to evaluate the cost effectiveness of prioritisation within our sample performed no better than a random ranking of the species. In contrast, prioritisation including management feasibility produced nearly two orders of magnitude improvement in cost effectiveness compared to a random ranking. We conclude that basing management actions on priorities based solely on risk assessment without considering management feasibility risks the inefficient use of limited resources. 

## Figures and Tables

**Table 1:** The top ten species identified by each of three prioritisation approaches: risk assessment (RA), risk management (RM) and both methods combined (RA + RM). For each approach, the cumulative cost for eradication of the species from GB in prioritised order is presented along with the individual RA (3 point ordinal scale; L = Low, M = Medium, H = High), RM (5 point ordinal scale; VL = Very Low, L = Low, M = Medium, H = High, VH = Very High) and RA + RM scores (5 point ordinal scale; VL = Very Low, L = Low, M = Medium, H = High, VH = Very High). (Original data on risk assessment and risk management scores from previously published studies [20,30,36]).

Prioritisation	Species	Cost to Eradicate	RA + RM Score	RM Score	RA Score
RM	*Tamias sibiricus*	£25,000	VH	VH	M
	*Procyon lotor*	£25,000	VH	VH	M
	*Corvus splendens*	£25,000	VH	VH	M
	*Procambarus acutus*	£125,000	H	VH	L
	*Threskiornis aethiopicus*	£25,000	VH	VH	M
	*Lacerta bilineata*	£600,000	H	VH	L
	*Nyctereutes procyonoides*	£125,000	H	H	M
	*Sarracenia purpurea*	£125,000	H	H	M
	*Orconectes limosus*	£600,000	H	H	M
	*Vespa velutina*	£600,000			
		£2,275,000			
RA	*Corbicula fluminalis*	£5,500,000	M	L	H
	*Hemigrapsus sanguineus*	£5,500,000	L	VL	H
	*Mnemiopsis leidyi*	£55,000,000	L	VL	H
	*Lysichiton americanus*	£55,000,000	M	L	H
	*Dreissena bugensis*	£55,000,000	L	VL	H
	*Rapana venosa*	£5,500,000	M	L	H
	*Procambarus clarkii*	£5,500,000	M	L	H
	*Ichthyosaura alpestris*	£5,500,000	H	M	H
	*Homarus americanus*	£600,000	M	L	M
	*Vespa velutina*	£600,000	H	M	M
		£193,700,000			
RA + RM	*Procyon lotor*	£25,000	VH	VH	M
	*Threskiornis aethiopicus*	£25,000	VH	VH	M
	*Tamias sibiricus*	£25,000	VH	VH	M
	*Corvus splendens*	£25,000	VH	VH	M
	*Sarracenia purpurea*	£125,000	H	H	M
	*Ichthyosaura alpestris*	£5,500,000	H	M	H
	*Lacerta bilineata*	£600,000	H	VH	L
	*Nyctereutes procyonoides*	£125,000	H	H	M
	*Procambarus acutus*	£125,000	H	VH	L
	*Orconectes limosus*	£600,000	H	H	M
		£7,175,000			

## Data Availability

RA and RM scores were available for all 26 species from previous studies, the methods and results of which are publicly available [20,30,36]. A copy of the dataset used in this analysis has been archived (Available on-line: http://doi.org/10.6084/m9.figshare.16940170 last accessed 1 December 2021).

## Supplementary Materials

The following are available online at https://www.mdpi.com/article/10.3390/biology10121320/s1.
